# Complete Genome Sequences of Two Mycobacterium smegmatis Bacteriophages

**DOI:** 10.1128/MRA.01329-20

**Published:** 2021-01-07

**Authors:** Darlenys Sanchez, Anna Acosta, Nicole Skoumpourdis, Dominican College SEA-PHAGES Annotators, Regina Alvarez, Bernadette J. Connors

**Affiliations:** a Department of Science, Dominican College of Blauvelt, Orangeburg, New York, USA; DOE Joint Genome Institute

## Abstract

Mycobacterium smegmatis, an acid-fast bacterial species in the phylum *Actinobacteria*, has often been used as a substitute for pathogenic mycobacteria in research. Here, we describe the isolation and characterization of two M. smegmatis bacteriophages, Penelope2018 and Miniwave.

## ANNOUNCEMENT

*Mycobacterium* is a genus of bacteria that contains the human pathogens M. tuberculosis and M. abscessus. With the evolution of multidrug-resistant mycobacteria, bacteriophages represent an alternative form of infection treatment (1). Using the nonpathogenic bacterial host M. smegmatis as a tool for phage discovery, a greater understanding of mycobacteriophage genome evolution can potentially broaden the implementation of phage therapies.

Mycobacteriophages Penelope2018 and Miniwave were directly isolated from untreated surface soil using the actinobacterial host M. smegmatis mc^2^155. Plaque purification, amplification, and creation of high-titer lysates were done using methods described in the Phage Discovery Guide (https://seaphagesphagediscoveryguide.helpdocsonline.com/home) (2). DNA was purified using the Promega Wizard DNA cleanup kit, and sequencing libraries were built using the Ultra II FS kit with dual-indexed barcoding (New England Biolabs). Libraries were then pooled and run on an Illumina MiSeq instrument to yield single-end 150-base reads. Genome quality assessment and assembly were completed using AceUtil and Newbler v.2.9 and checked for completeness, accuracy, and genomic termini using Consed v.29 (3) with default parameters as previously described (4) (https://github.com/SEA-PHAGES/phageAssembler). The genomes were manually annotated on PECAAN (5) and DNAMaster v.5.0.2 (http://cobamide2.bio.pitt.edu/computer.htm) using Glimmer v.3.02 (6), GeneMark v.2.8 (7), and Starterator (https://github.com/SEA-PHAGES/starterator) to determine start sites. ARAGORN v.1.1 and tRNAscan v.2.0 were used to detect tRNA (8, 9). Putative functions were determined using BLASTp on the NCBI nonredundant (nr) and actinobacteriophage databases (E value of <10e^−7^ or less), as well as HHPRED (probability value of >90%) (10–12). Genome and phage features can be found in Table 1.

**TABLE 1:** Mycobacteriophage genome details

Phage name	GenBank accession no.	SRA accession no.	Genome length (bp)	G+C content (%)	No. of CDS^*a*^	Collection site location	Plaque and TEM images available at:
Penelope2018	MN119378	SRX9125697	64,544	59.7	88	41.2295 N, 73.9871 W	https://phagesdb.org/phages/Penelope2018
Miniwave	MT952854	SRX9537687	74,961	63	139	41.2295 N, 73.9871 W	https://phagesdb.org/phages/Miniwave

a CDS, coding DNA sequences.

Penelope2018 plaques are not turbid. Sequencing produced 686,783 individual reads with approximately 1,509-fold shotgun coverage. The genome displays 99% nucleotide similarity to BigMama, as determined by a BLASTn search on the NCBI nr database. Penelope2018 contains a circularly permuted genome of 64,544 bp and a G+C content of 59.7%. Annotation revealed 88 open reading frames (ORFs), 23 of which have predicted functions, only 3 of which are coded on the reverse strand. These features are characteristic of phages in the D1 subcluster (13), which contains 19 sequenced mycobacterial phages in the SEA-PHAGES collection (11).

Miniwave plaques are turbid, indicating that the phage is temperate. Sequencing produced 795,539 individual reads with approximately 152-fold shotgun coverage. This genome displays 99% nucleotide similarity to Paperbeatsrock, as determined by a BLASTn search on the NCBI nr database. It has a genome of 64,544 bp and a G+C content of 63% with 3′ sticky 10-bp overhang genomic ends. Miniwave has 139 ORFs, 43 of which have predicted functions and no predicted tRNAs. These characteristics are expected of phages in the E cluster (14), which contains 106 sequenced mycobacterial phages in the SEA-PHAGES collection, with no identified subclusters (11).

Phage particles were freshly prepared from a high-titer lysate for transmission electron microscopy (TEM). Particles were negatively stained with 2% (wt/vol) aqueous uranyl acetate (pH 4.5) on carbon-coated grids and examined at an accelerating voltage of 100 kV (images available on phagesdb.org). Both Miniwave and Penelope2018 are siphoviruses, which agrees with other mycobacterial phages in the D1 and E clusters.

### Data availability.

The genome sequences reported here have been deposited in GenBank under the accession numbers in Table 1 (BioProject PRJNA488469). The SRA numbers are provided in Table 1.
